# Mice Lacking the SLAM Family Member CD84 Display Unaltered Platelet Function in Hemostasis and Thrombosis

**DOI:** 10.1371/journal.pone.0115306

**Published:** 2014-12-31

**Authors:** Sebastian Hofmann, Attila Braun, Rastislav Pozgaj, Martina Morowski, Timo Vögtle, Bernhard Nieswandt

**Affiliations:** University of Würzburg, Department of Experimental Biomedicine, University Hospital and Rudolf Virchow Center, Würzburg, Germany; National Cerebral and Cardiovascular Center, Japan

## Abstract

**Background:**

Platelets are anuclear cell fragments derived from bone marrow megakaryocytes that safeguard vascular integrity by forming thrombi at sites of vascular injury. Although the early events of thrombus formation—platelet adhesion and aggregation—have been intensively studied, less is known about the mechanisms and receptors that stabilize platelet-platelet interactions once a thrombus has formed. One receptor that has been implicated in this process is the signaling lymphocyte activation molecule (SLAM) family member CD84, which can undergo homophilic interactions and becomes phosphorylated upon platelet aggregation.

**Objective:**

The role of CD84 in platelet physiology and thrombus formation was investigated in CD84-deficient mice.

**Methods and Results:**

We generated CD84-deficient mice and analyzed their platelets *in vitro* and *in vivo*. *Cd84^−/−^* platelets exhibited normal activation and aggregation responses to classical platelet agonists. Furthermore, CD84 deficiency did not affect integrin-mediated clot retraction and spreading of activated platelets on fibrinogen. Notably, also the formation of stable three-dimensional thrombi on collagen-coated surfaces under flow *ex vivo* was unaltered in the blood of *Cd84^−/−^* mice. *In vivo*, *Cd84^−/−^* mice exhibited unaltered hemostatic function and arterial thrombus formation.

**Conclusion:**

These results show that CD84 is dispensable for thrombus formation and stabilization, indicating that its deficiency may be functionally compensated by other receptors or that it may be important for platelet functions different from platelet-platelet interactions.

## Introduction

Platelets are essential players in thrombosis and hemostasis and “survey” the integrity of the vascular system by discriminating between intact or injured vessel walls [1]. Upon damage of the endothelial cell lining, platelets rapidly adhere to components of the newly exposed subendothelial extracellular matrix (ECM), e.g. collagen. Subsequently, they become activated and initiate a self-amplifying feedback-loop, resulting in enhanced platelet activation and recruitment of additional platelets from the circulation. Finally, the complex interaction between platelets, the ECM and blood components leads to the formation of a stable thrombus that seals the wound. Under pathological conditions, however, excessive thrombus formation may result in vessel occlusion and subsequently lead to myocardial infarction or ischemic stroke [2], [3].

A key event in the process of thrombus formation is the activation of integrin αIIbβ3, which bridges adjacent platelets and mediates stable platelet adhesion to the ECM by binding to fibrinogen, fibronectin, von Willebrand factor (vWF) and multiple ECM proteins [3], [4]. Outside-in signaling through the integrin further enhances aggregation [5], but also additional receptors on the platelet surface, as well as soluble mediators, are required for stable aggregation [6]. Among these molecules is CD40L which, upon release from the platelet surface, supports stable formation of arterial thrombi by binding to integrin αIIbβ3 [7]. The close proximity of platelets within the aggregates allows contact-dependent signaling via interactions of receptors with their ligands on adjacent plasma membranes, like junctional adhesion molecules (JAMs) [8] and ephrins/Eph kinases [9].

CD84, a member of the signaling lymphocyte activation molecule (SLAM) family, is expressed on the surface of platelets and is also implicated to stabilize thrombi via homophilic interactions, but experimental evidence to support this hypothesis has not been provided so far [10], [11]. Members of the SLAM family are well recognized as important immunomodulatory receptors and are expressed on the surface of a wide variety of hematopoietic cells [12]. CD84 is a type I transmembrane glycoprotein with an N-terminal ectodomain that comprises a membrane-proximal Ig constant domain and a membrane-distal Ig variable domain [13], which mediates the homophilic interaction between CD84 proteins [14], [15]. The C-terminal intracellular portion of CD84 bears two immunoreceptor tyrosine-based switch motifs (ITSM), which can bind the intracellular adapters SLAM-associated protein (SAP also termed SH2D1A) and Ewing's sarcoma activated transcript 2 (EAT-2) [10], [16], [17].

Ligation of CD84 with a monoclonal antibody results in phosphorylation of the ITSMs and subsequent SAP recruitment [10], [16], resulting in enhanced IFNγ production and proliferation in T cells stimulated with low doses of anti-CD3 antibody [14], [16]. A study in CD84-deficient mice established CD84 as a functional co-receptor in lymphocytes that facilitates prolonged B cell:T cell interaction required for optimal germinal center formation [18].

The effect of CD84-deficiency on platelet function has not been analyzed to date. However, several findings implicate that CD84 and its downstream signaling pathway may be of relevance in platelets. First, the cytoplasmic tail of CD84 is phosphorylated in response to platelet aggregation or upon antibody-mediated receptor crosslinking. Second, wild-type platelets, but not SAP-deficient platelets, are able to spread on immobilized CD84 [10]. Therefore, CD84 has been proposed to mediate contact-dependent signaling and contribute to thrombus stabilization [10]. Third, a recent study from our laboratory demonstrated that CD84 receptor levels on platelets are tightly regulated by two distinct and independent proteolytic mechanisms upon platelet activation: shedding of the extracellular part by a disintegrin and metalloproteinase (ADAM) 10 and cleavage of the intracellular C-terminus by the protease calpain [19].

## Material and Methods

### Mice

Generation of CD84-deficient mice: A targeting vector was designed to replace a genomic region comprising the splice donor site of *Cd84* exon 1, the complete intron 1, and a critical part of exon 2 (comprising parts of the 5′UTR, the translation initiation site and the coding sequence of the CD84 signal peptide) by a cassette containing a neomycin resistance gene. The homologous arms flanking this cassette facilitate site specific recombination. The targeting vector was purified and electroporated into R1 embryonic stem (ES) cells derived from the 129/Sv mouse strain [20]. After Geneticin (G418) selection, targeted stem cell clones were screened by Southern blot analysis with a gene-specific external probe. *Cd84^+/−^* ES cells were injected into C57BL/6 blastocysts to generate chimeric mice. Male chimeras were backcrossed with C57BL/6 mice (Harlan Laboratories) and subsequent progeny were intercrossed to obtain *Cd84^−/−^* mice. Genotypes were determined by Southern blot analysis and PCR. Animal studies were approved by the district government of Lower Franconia (Bezirksregierung Unterfranken).

### Chemicals and reagents

Collagen (Kollagenreagent Horm; Nycomed), convulxin (Enzo Lifesciences) α-thrombin (Roche Diagnostics), adenosine diphosphate (ADP), sodium heparin, human fibrinogen, apyrase type III, prostacyclin (PGI_2_), Igepal CA-630 (all from Sigma-Aldrich), U46619 (Alexis Biochemicals) and ECL solution (PerkinElmer) were purchased, collagen-related peptide (CRP) was generated as described [21]. Rhodocytin was a generous gift from Prof. Dr. J. Eble (Münster University Hospital, Germany). The anesthetic drugs medetomidine (Pfizer), midazolam (Roche Pharma AG), and fentanyl (Janssen-Cilag GmbH) and the antagonists atipamezol (Pfizer), flumazenil, and naloxon (both from Delta Select GmbH) were used according to the regulation of the local authorities. The antibody against the activated form of integrin αIIbβ3 (JON/A-PE) was from Emfret Analytics. Anti-murine CD84 monoclonal antibody JER1 [19] and other antibodies were generated and modified in our laboratories as described [22].

### Platelet preparation

Mice were bled under isoflurane anesthesia from the retro-orbital plexus. Blood was collected in a tube containing heparin in tris-buffered saline (TBS) (20 U/mL pH 7.3), and platelet-rich plasma (prp) was obtained by two cycles of centrifugation at 300 g for 5 minutes at room temperature (RT). For preparation of washed platelets, prp was washed twice at 800 g for 5 minutes at RT by resuspending the pellet in modified Tyrode's-HEPES buffer (137 mM NaCl, 0.43 mM Na_2_HPO_4_, 12 mM NaHCO_3_, 2.7 mM KCl, 1 mM MgCl_2_, 20 mM HEPES, 0.1% glucose, 0.35% bovine serum albumin, pH 7.3) in the presence of PGI_2_ (0.1 µg/mL) and apyrase (0.02 U/mL). Platelets were then resuspended in Tyrode's-HEPES buffer containing 0.02 U/mL apyrase and 0 mM or 2 mM CaCl_2,_ depending on the experiment.

### Platelet aggregometry

Washed platelets were prepared as described above. 50 µL platelet suspension (0.5×10^6^ platelets/µL) was mixed with 110 µL Tyrode's-HEPES buffer containing 2 mM CaCl_2_ and 100 µg/mL fibrinogen. Aggregation was triggered by the indicated platelet agonists and changes in light transmission were recorded on a Fibrintimer 4 channel aggregometer (APACT Laborgeräte und Analysensysteme). Buffer represents 100% transmission. Aggregation curves for thrombin were obtained in the absence of fibrinogen, measurements with ADP were performed in prp.

### Clot retraction

For clot retraction studies, 250 µL prp, adjusted to a concentration of 0.3×10^6^ platelets/µL, were mixed with 1 µL erythrocyte suspension and supplemented with CaCl_2_ to a final concentration of 20 mM. Clotting was induced by addition of high thrombin concentrations (5 U/mL). Subsequent clot retraction was monitored at 37°C under non-stirring conditions and documented with a digital camera at different time points.

### Platelet spreading assay

Coverslips were coated with 200 µg/mL human fibrinogen overnight and blocked with PBS containing 1% BSA. After rinsing with Tyrode's-HEPES buffer, washed platelets prestimulated with 0.01 U/mL thrombin were added and incubated at RT for the indicated time periods. Bound platelets were fixed with 4% PFA, the coverslips were rinsed again and platelets were visualized with a Zeiss Axiovert 200 inverted microscope (x100).

### Western blot

Platelets were washed twice in Tyrode's-HEPES buffer and lysed with immunoprecipitation buffer containing 1% Igepal CA-630. After 10 min centrifugation at 14000 rpm for 10 min at 4°C the supernatant was obtained, mixed with the respective amount of 4x SDS sample buffer and boiled for 5 min at 95°C.

Proteins were separated by sodium dodecyl sulfate polyacrylamide gel electrophoresis (SDS-PAGE) and blotted onto polyvinylidene difluoride membranes. After blocking with 5% fat-free milk in TBS-T, the membrane was incubated with peroxidase-conjugated monoclonal antibody JER1 at 4°C overnight. Bound antibody was visualized by ECL.

### Flow cytometry

Whole blood was diluted 1∶20 in Tyrode's-HEPES buffer, incubated with appropriate fluorophore-conjugated monoclonal antibodies for 15 min at RT and analyzed on a FACSCalibur instrument (Becton Dickinson, Heidelberg, Germany) to determine glycoprotein expression. Parallel analyses on platelet count and size were performed with an automated cell analyzer (Sysmex, Norderstedt, Germany).

To analyze platelet activation responses, washed blood was activated with agonists at the indicated concentrations and stained with fluorophore-conjugated monoclonal antibodies at saturating concentrations for 14 minutes at 37°C and analyzed.

### Platelet life span

Circulating platelets were labeled *in vivo* by intravenous injection of 5 µg Dylight-488-anti-GPIX Ig derivative in 200 µL PBS in the retro-orbital plexus. 30 min after antibody injection (and every 24 h for 5 days) 50 µL blood were taken from the retro-orbital plexus of treated mice and the percentage of the Dylight-488-positive population was determined by flow cytometry.

### Adhesion under flow

Heparinized whole blood was perfused over collagen-coated cover slips as described [23] at shear rates of 1000 s^−1^, 1700 s^−1^ and 3000 s^−1^. Before perfusion, anti-coagulated blood was incubated with DyLight-488–conjugated anti-GPIX Ig derivative (0.2 µg/mL) at 37°C for 5 minutes. Aggregate formation was visualized with a Zeiss Axiovert 200 inverted microscope (40x/0.60 objective). Phase-contrast and fluorescence pictures were recorded with a CoolSNAP-EZ camera, and analyzed off-line using MetaVue software. Thrombus formation was analyzed as the mean percentage of the total area covered by platelets/thrombi in phase contrast images. Mean integrated fluorescence intensity per mm^2^ represented a measure of thrombus volume.

### Tail bleeding time assay

Mice were anesthetized with a triple anesthesia (medetomidine 0.5 µg/g, midazolam 5 µg/g and fentanyl 0.05 µg/g body weight) and a 1 mm segment of the tail tip was removed with a scalpel. Tail bleeding was monitored by gently absorbing the drop of blood with a filter paper in 20 s intervals without directly contacting the wound site. When no blood was observed on the paper, bleeding was determined to have ceased. The experiment was manually stopped after 20 min by cauterization [24].

### Intravital microscopy of FeCl_3_-injured mesenteric arterioles

Mice (4–5 weeks of age, weight 15–18 g) were anesthetized and the mesentery was exteriorized through a midline abdominal incision. Arterioles were visualized with a Zeiss Axiovert 200 inverted microscope (10x objective) equipped with a 100-W HBO fluorescent lamp source and a CoolSNAP-EZ camera (Visitron, Munich, Germany). Digital images were recorded and analyzed off-line using MetaVue software. Injury was induced by topical application of a 3 mm^2^ filter paper saturated with FeCl_3_ (20%). Adhesion and aggregation of fluorescently labeled platelets (Dylight-488-conjugated anti-GPIX Ig derivative) in arterioles was monitored for 40 min or until complete occlusion occurred (blood flow stopped for >1 min).

### Mechanical injury of the abdominal aorta

The abdominal cavity of anesthetized mice (∼6 weeks of age) was opened by a longitudinal incision and the abdominal aorta was exposed. A Doppler ultrasonic flow probe (Transonic Systems, New York, USA) was placed around the aorta and a mechanical injury was induced by a single firm compression with forceps upstream of the flow probe. Blood flow was monitored until complete thrombotic occlusion of the aorta occurred, or up to 30 min [25].

### Statistics

Results from at least three independent experiments per group are presented as mean ± standard deviation (SD). Differences between two groups were statistically analyzed using a modified t-test (Welch's test). *p*-values <0.05 compared to control were considered statistically significant (*p*-value <0.05 = *; <0.01 = **; <0.001 = ***).

## Results

### Generation and phenotype of CD84-deficient mice

**Figure 1:**
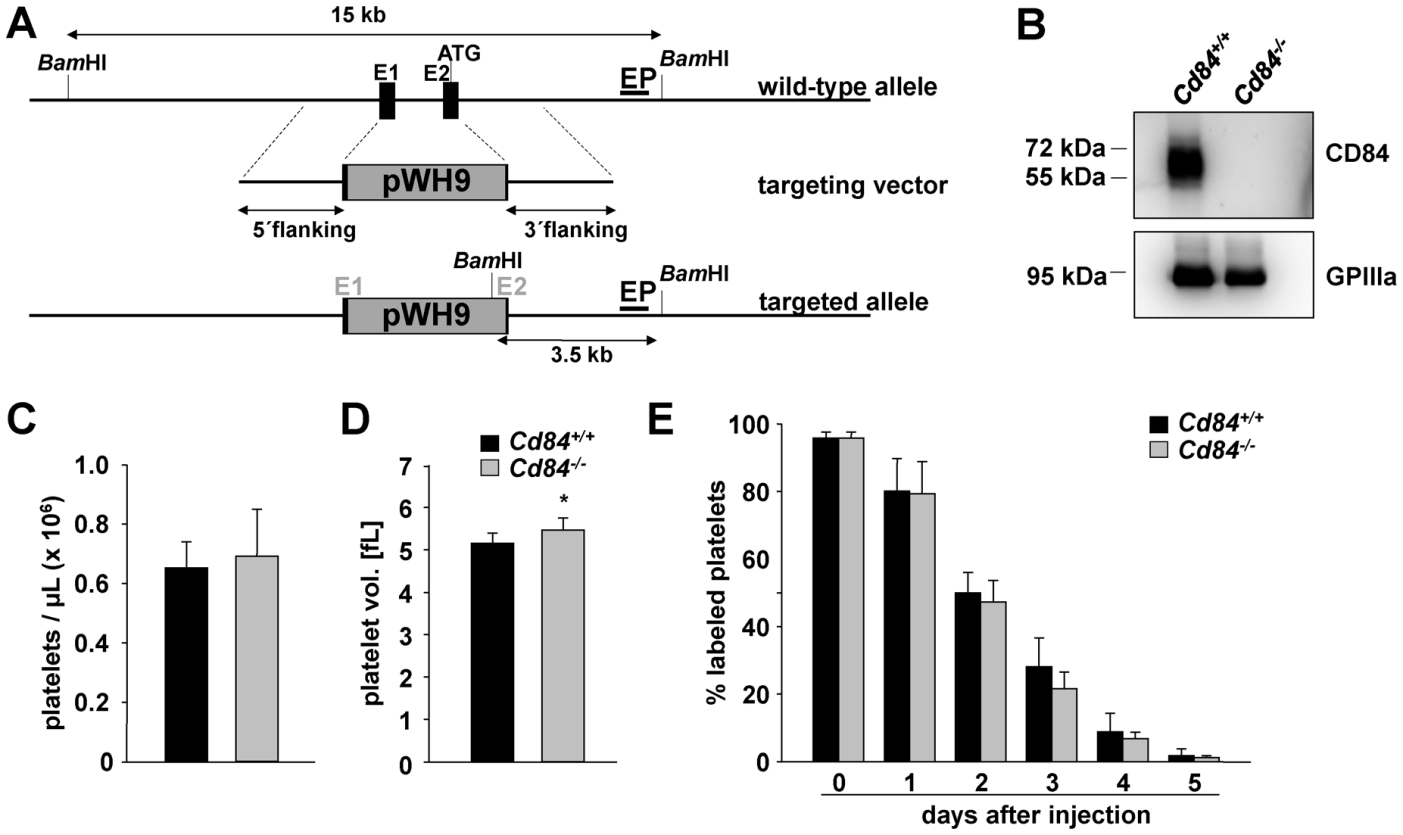
CD84-deficient mice display normal platelet count and size. (A) CD84 targeting strategy: This scheme illustrates the detection of wild-type and *Cd84^−/−^* (targeted) alleles. Upon homologous recombination, the pWH9 cassette containing a neomycin resistance gene disrupts the *Cd84* gene. An external probe (EP) recognizes a sequence downstream of 3′ arm in intron 2. With the pWH9 cassette, a new *Bam*HI restriction site is introduced, enabling the determination of a wild-type and *Cd84^−/−^* band by Southern blot analysis. (B) Analysis of CD84 expression in wild-type (*Cd84^+/+^*) and *Cd84^−/−^* platelets by Western blot. Expression of GPIIIa was used as loading control. (C) Peripheral platelet counts and (D) platelet volume of wild-type and *Cd84^−/−^* mice measured with a blood cell counter. (E) Determination of the platelet life span in wild-type and *Cd84^−/−^* mice. Mice were injected with a DyLight 488-conjugated anti-GPIX Ig derivate to label platelets *in vivo*. Results are % of fluorescently labeled platelets at the indicated days after injection as determined by flow cytometry. Values are mean ± SD of 5 mice per group.

Since CD84 expression is known to be restricted mainly to the hematopoietic system, we generated constitutive knockout mice (Fig. 1A). To this end, we designed a vector to replace parts of exon 1, the complete intron 1 and a critical part exon 2 by a cassette containing a neomycin resistance gene allowing for selection of recombinant clones. Homologous arms facilitate site specific recombination. By means of this strategy, the 5′ UTR of the mRNAs and the coding sequence of the signal peptide are deleted. Deletion of the 5′ UTR of the mRNA causes inhibition of ribosome binding and therefore protein synthesis is abolished. If still alternatively spliced CD84 isoforms would be translated from mRNAs, these isoforms without signal peptide would not be transported to the plasma membrane in the cells. The targeting vector was electroporated into 129/Sv-derived ES cells, and ES cells with successful homologous recombination were injected into C57BL/6 blastocysts. Germ line transmission of the targeted allele was obtained by crossing the resulting chimeric mice with C57BL/6 mice to generate *Cd84^+/−^* mice, which were then intercrossed to produce *Cd84^−/−^* mice. The expression of CD84 in wild-type (*Cd84^+/+^*) platelets and its absence in platelets from *Cd84^−/−^* mice was confirmed by Western blot analysis (Fig. 1B) and flow cytometry (data not shown). *Cd84^−/−^* mice were born at Mendelian ratios, developed normally and were grossly indistinguishable from wild-type mice (data not shown), in line with a previous study [18]. Analysis of basic blood parameters with a Sysmex cell counter revealed unaltered white and red blood cell counts in *Cd84^−/−^* mice compared to wild-type controls (Table 1). Platelet count, life span and the expression of prominent platelet surface proteins were unaltered in *Cd84^−/−^* mice compared to wild-type controls (Fig. 1C,E and Table 1). However, *Cd84^−/−^* platelets showed a modest increase in size (Fig. 1D). Taken together, these data indicate that CD84 is dispensable for the development of the hematopoietic system.

**Table 1:** *Cd84^−^* ^/*−*^ mice display normal hematologic parameters and unaltered levels of platelet surface glycoproteins.

A			
	Cd84^+/+^	Cd84*^−^* ^/*−*^	p
**WBC×10^3^/µL**	6.30±2.66	6.58±3.05	n.s.
**RBC×10^3^/µL**	8.33±0.85	7.90±1.56	n.s.
**HGB [g/dL]**	13.93±1.30	12.74±2.21	n.s.
**HCT [%]**	44.03±3.78	41.37±6.83	n.s.

B			
	MFI ± SD *Cd84^+/+^*	MFI ± SD *Cd84^−/−^*	*p*
**GPIb**	326±29	328±20	n.s.
**GPIX**	439±17	459±19	n.s.
**GPV**	292±10	299±15	n.s.
**CD9**	1334±43	1305±64	n.s.
**GPVI**	32±5	41±+7	n.s.
**αIIbβ3**	462±49	469±24	n.s.
**α2**	54±3	55±3	n.s.
**β1**	152±16	154±6	n.s.
**CLEC-2**	112±6	114±7	n.s

(A) White blood cell (WBC) count, red blood cell (RBC) count, hemoglobin (HGB) and hematocrit (HCT) were determined with a hematologic analyzer (Sysmex) (n = 5, two independent experiments, n.s.  =  not significant). (B) Expression of glycoproteins on the platelet surface was determined by flow cytometry. Diluted whole blood from the indicated mice was incubated with FITC-labeled antibodies at saturating conditions for 15 minutes at RT, and platelets were analyzed directly. Data are expressed as mean fluorescence intensity ± SD (n = 4) and are representative of 3 individual experiments.

### Unaltered function of *Cd84^−/−^* platelets *in vitro*

**Figure 2:**
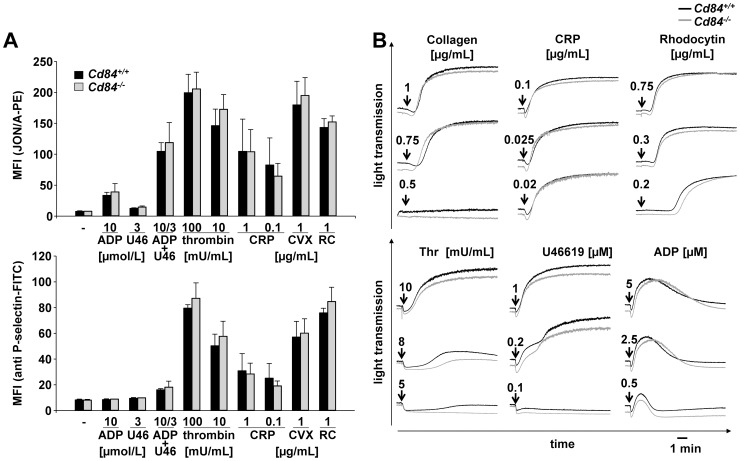
Normal αIIbβ3 activation, α-granule release and aggregation response of *Cd84^−/−^* platelets. (A) Flow cytometric analysis of integrin αIIbβ3 activation (upper panel) and degranulation-dependent P-selectin exposure (lower panel) in response to the indicated agonists in wild-type and *Cd84^−/−^* platelets. Results are mean fluorescence intensities (MFI) ± SD of 4 mice per group and are representative of 4 individual experiments. CRP: collagen-related peptide, CVX: convulxin, and RC: rhodocytin. (B) Washed platelets from wild-type (black line) and *Cd84^−/−^* (gray line) mice were activated with the indicated agonist concentrations and light transmission was recorded on a Fibrintimer 4-channel aggregometer. ADP measurements were performed in prp. Representative aggregation traces of at least 3 individual experiments are depicted (for RC: n = 4 mice per group).

To determine whether the lack of CD84 had functional consequences on platelet activation, platelets of wild-type and CD84-deficient animals were stimulated with different agonists and analyzed by flow cytometry. Activation of integrin αIIbβ3, as assessed by binding of the JON/A-PE antibody, and degranulation-dependent P-selectin surface exposure were used as markers for platelet activation. *Cd84^−/−^* platelets showed an unaltered response towards the G protein-coupled receptor (GPCR) agonists thrombin, ADP and the stable thromboxane A_2_ (TxA_2_) analogue U46619 (Fig. 2A). Similarly, platelet activation via the immunoreceptor tyrosine-based activation motif (ITAM)-coupled receptor glycoprotein (GP) VI [26] by collagen or CRP and via the (hem)ITAM receptor C-type lectin-like receptor 2 (CLEC-2) [27] by the snake venom toxin rhodocytin (RC) was not affected by the loss of CD84 (Fig. 2A).

CD84 has been shown to become tyrosine phosphorylated upon platelet aggregation, and therefore may act as an aggregation-induced co-receptor supporting stable aggregate formation [10]. To test this directly, we performed *in vitro* aggregation studies with washed platelets from wild-type and *Cd84^−/−^* mice. *Cd84^−/−^* platelets showed unaltered shape change and the aggregometry light transmission traces showed similar maximal aggregation rates as for wild-type platelets in response to all tested agonists (collagen, CRP, thrombin, U46619, ADP, RC). Also at intermediate and low agonist concentrations no significant alteration of aggregation was detectable (Fig. 2B). Thus, *in vitro* aggregation results indicated that the loss of CD84 does not affect aggregate formation or stability, at least under the experimental conditions used.

**Figure 3:**
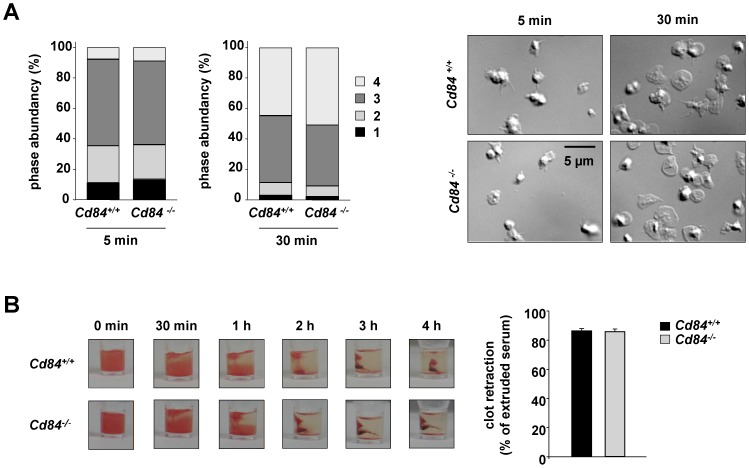
Normal integrin outside-in signaling in *Cd84^−/−^* platelets. (A) Washed platelets of wild-type and *Cd84^−/−^* mice were allowed to spread on fibrinogen (100 µg/mL) for 30 min after stimulation with 0.01 U/mL thrombin. Statistical evaluation of the percentage of spread platelets at different spreading stages and representative differential interference contrast (DIC) images of 2 individual experiments. Spreading stages: 1: roundish, 2: only filopodia, 3: filopodia and lamellipodia, 4: fully spread (scale bar 5 µm). (B) Clot retraction of *Cd84^+/+^* and *Cd84^−/−^* platelets in prp upon activation with 5 U/mL thrombin in the presence of 20 mM CaCl_2_ at the indicated time points (n = 6, left) and extruded serum after clot formation (right). Values are mean ± SD.

Aggregation of platelets requires inside-out as well as outside-in signaling of integrins. Another process requiring integrin αIIbβ3-mediated adhesion, outside-in signaling and subsequent rearrangements of the cytoskeleton is spreading of platelets on fibrinogen [28]. To study the role of CD84 in this process, washed platelets were pre-stimulated with 0.01 U/mL thrombin and allowed to spread on a fibrinogen-coated surface. *Cd84^−/−^* platelets formed filopodia and lamellipodia to the same extent and with similar kinetics as wild-type platelets resulting in ∼50% fully spread platelets after 30 min in both groups (Fig. 3A). Integrin αIIbβ3 outside-in signaling also regulates clot retraction [29]. In the process of clot retraction, platelets generate force to contract the fibrin mesh, decrease the clot size, and pull together the edges of damaged tissue to form a mechanically stable clot [30]. To study whether the loss of CD84 alters clot retraction, clot formation was induced in prp of *Cd84^−/−^* and wild-type mice by addition of a high dose of thrombin (5 U/mL) in the presence of 20 mM Ca^2+^ and clot retraction was monitored over time. No differences between wild-type and *Cd84^−/−^* platelets were observed and the excess fluid extruded after clot retraction was similar in both groups (Fig. 3B). Hence, absence of CD84 does not significantly influence the ability of platelets to undergo integrin αIIbβ3-mediated adhesion and to perform the reorganization of the actin cytoskeleton to mediate shape change, spreading and clot retraction.

### Unaltered aggregate formation under flow *ex vivo* in *Cd84^−/−^* blood

**Figure 4:**
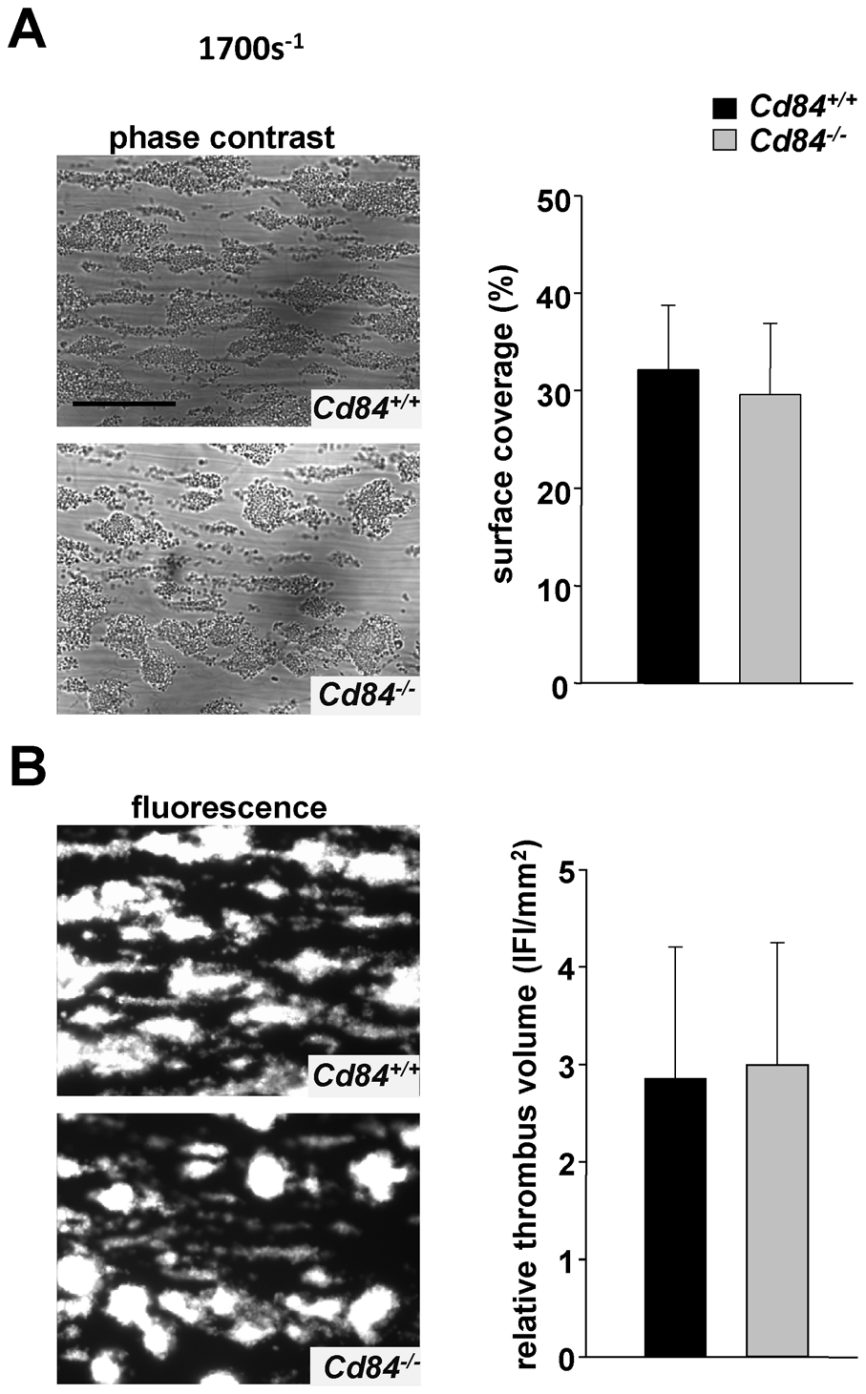
Normal adhesion and aggregate formation of *Cd84^−/−^* platelets on collagen under flow. (A) Whole blood from *Cd84^+/+^* or *Cd84* ^−*/*−^ mice was perfused over a collagen-coated surface (0.2 mg/mL) at a shear rate of 1700 s^−1^. Representative phase contrast images of aggregate formation on collagen after 4 minutes of perfusion time (scale bar 50 µm) (left) and mean surface coverage (right). (B) Representative fluorescence images on aggregate formation on collagen after 4 minutes of perfusion time (left) and relative thrombus volume expressed as integrated fluorescence intensity (IFI) ± SD of n = 5 mice per group (right).

In contrast to the *in vitro* situation, the prevailing shear forces as well as the rapid dilution of second wave mediators in flowing blood critically influence platelet aggregation and thrombus formation at sites of vascular injury. Under such conditions, the lack of receptors that potentially modify platelet aggregate stability could become functionally evident. To test the consequence of CD84-deficiency on aggregate formation under flow, anti-coagulated whole blood was perfused over a collagen-coated surface in an *ex vivo* flow chamber system, at high (1700 s^−1^) and intermediate (1000 s^−1^) shear rates (Fig. 4 and data not shown). Similar to wild-type controls, platelets from CD84-deficient mice rapidly adhered to collagen and formed three-dimensional aggregates at both tested shear rates. Evaluation of surface coverage (Fig. 4A) and relative thrombus volume (Fig. 4B) at the end of the 4-minute observation period did not reveal statistically significant differences between *Cd84* ^−*/*−^ and wild-type mice. At very high shear rates (3000 s^−1^), wild-type and CD84-deficient mice only formed very small thrombi but showed similar surface coverage (data not shown). These findings indicate that CD84 is not essential for growth and stabilization of platelet-rich thrombi under intermediate and high shear *ex vivo*.

### Normal hemostasis and arterial thrombus formation in *Cd84^−/−^* mice

**Figure 5:**
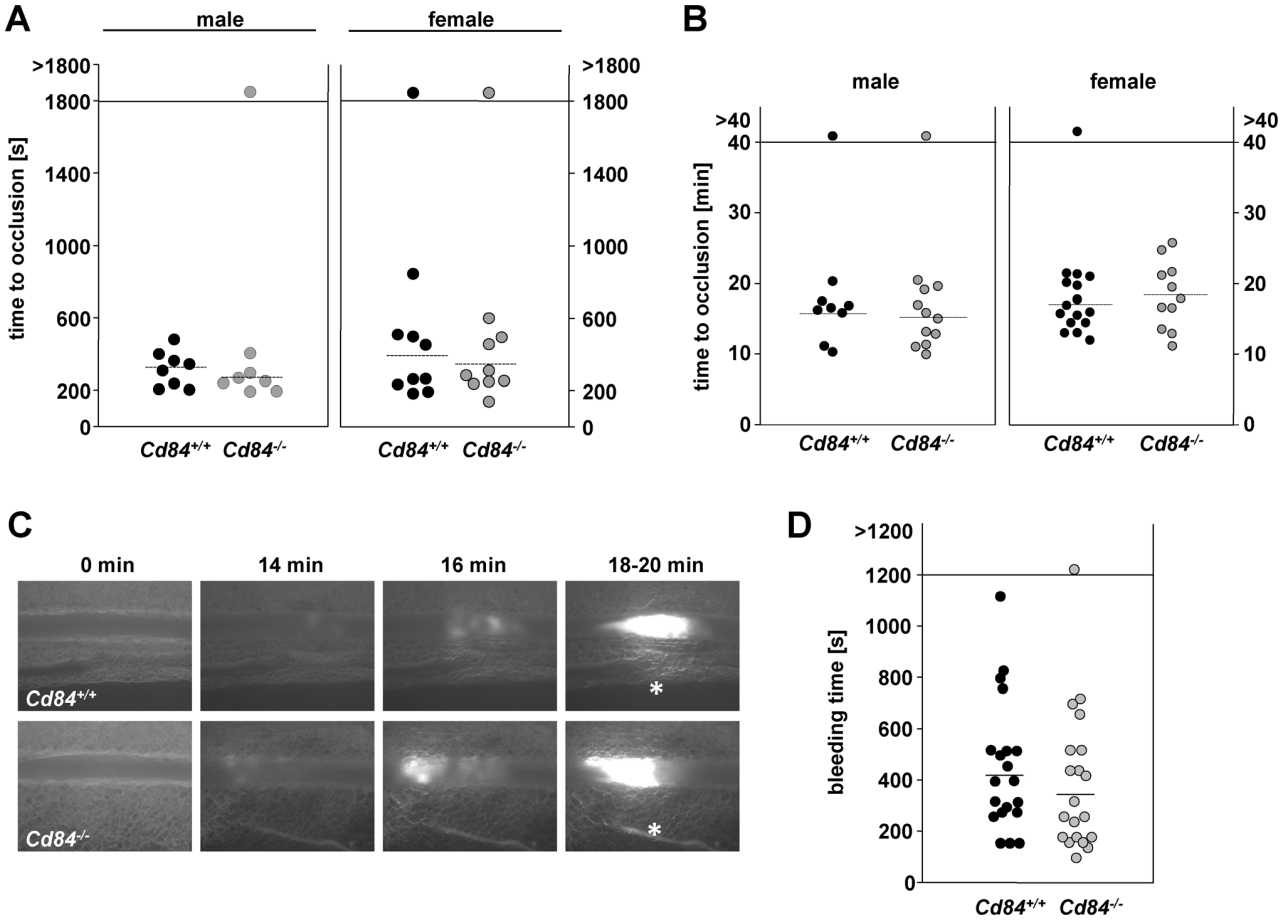
Unaltered thrombotic and hemostatic function in Cd84-deficient mice. (A) The abdominal aorta was injured by firm compression with a forceps and blood flow was monitored for 30 min. Each symbol represents one animal. (B) Small mesenteric arterioles were injured by topical application of FeCl_3_ and occlusive thrombus formation was monitored using intravital microscopy. Each symbol represents one mesenteric arteriole. The horizontal dotted line indicates the mean time to vessel occlusion. (C) Representative images of the FeCl_3_-induced injury model of mesenteric arterioles in *Cd84^+/+^* and *Cd84^−^* ^/*−*^ mice, asterisk indicates stable occlusion of the vessel. (D) 1 mm tail tip was amputated and tail bleeding times of *Cd84^+/+^* and *Cd84^−/−^* mice were monitored. Each symbol represents one animal. The horizontal dotted line indicates the mean time to vessel occlusion.

To study the effect of CD84 deficiency on arterial thrombus formation *in vivo*, we subjected mice to a thrombosis model in which the abdominal aorta is mechanically injured and blood flow is monitored with an ultrasonic perivascular Doppler flow probe. Female and male *Cd84* ^−*/*−^ mice were tested separately to explore whether sex-specific factors may lead to divergent results, as described for SLAM (CD150)-deficient mice [10]. In line with our *in vitro* results, wild-type and *Cd84^−/−^* mice of both gender formed occlusive thrombi with comparable kinetics (mean occlusion time: male *Cd84^+/+^*: 325±99 s vs. male *Cd84^−/−^*: 271±72 s; female *Cd84^+/+^*: 392±217 s vs. female *Cd84^−/−^*: 345±148 s, Fig. 5A). Similarly, the mean time to occlusion in a model of FeCl_3_-induced injury of mesenteric arterioles was comparable between wild-type and *Cd84^−/−^* mice for males and females (mean time to occlusion: male *Cd84^+/+^*: 15.73±3.32 min vs. male *Cd84^−/−^*: 15.19±3.69 min; female *Cd84^+/+^*: 17.03±3.15 min vs. female *Cd84^−/−^*: 18.46±4.78 min, Fig. 5B). Representative images of thrombus formation in this model are shown in Fig. 5C.

To test whether CD84 deficiency affects hemostasis, tail bleeding times were determined (Fig. 5D). Time until arrest of bleeding was not significantly altered in CD84-deficient mice (*Cd84^+/+^*: 418±206 s; *Cd84^−/−^*: 347±201 s). When data for female and male mice were analyzed separately, there was neither a significant difference between wild-type and *Cd84^−/−^* males nor between wild-type and *Cd84^−/−^* females (data not shown). Taken together, these results indicate that CD84 is not required for hemostatic and thrombotic function of platelets *in vivo*.

## Discussion

In this study we used CD84-deficient mice to assess the role of this SLAM family member for platelet function *in vitro* and *in vivo*. We show that the lack of CD84 in platelets does not affect classical platelet functions such as integrin activation, granule release and aggregation in response to major agonists or spreading *in vitro*. *Cd84^−/−^* mice showed unaltered bleeding times and normal arterial thrombus formation *in vivo* after experimental injuries indicating that CD84 does not serve an essential function in hemostatic/thrombotic processes.

CD84 expression in platelets has been reported in earlier studies [10], [31], but its role in platelet activation and thrombus formation has been elusive. A previous study revealed that CD84 undergoes tyrosine phosphorylation upon platelet activation and aggregation [10]. One of the two phosphorylated cytoplasmic tyrosines was found in an ITSM, which is a putative recognition motif for the adapter proteins SAP and EAT-2. The requirement of SAP for platelet spreading on immobilized CD84 implicates a functional relevance of this signaling pathway. Interestingly, activation-induced tyrosine phosphorylation of CD84 was abolished when platelet aggregation was blocked with an αIIbβ3 inhibitor [10]. This aggregation-dependent phosphorylation was also observed for the other prominent SLAM family member on platelets, CD150. The subsequent analysis of CD150-deficient female mice further revealed a delay in thrombus formation in a FeCl_3_-induced thrombosis model in mesenteric arteries and weaker aggregation in response to collagen and a thrombin receptor activating peptide [10]. Due to these results CD150 and CD84 were proposed as thrombus stabilizing receptors in response to platelet aggregation but CD84-deficient mice were not available at that time to confirm this. However, a potential thrombus-stabilizing function of SLAM family members in platelets was further supported by the cooperation of CD84 and Ly108 in the stabilization of T cell:B cell contacts [18]. In contrast, homophilic interaction of CD84 has been shown to negatively regulate FcεRI-ITAM signaling in mast cells [32], which was found to be independent of SAP and EAT-2, but dependent on the inhibitory kinase Fes [33]. Hence, also a negative regulatory role for CD84 in thrombus formation would have been conceivable.

The current study provides the first analysis of CD84-deficient platelets. Besides a slightly elevated platelet size, *Cd84^−/−^* mice display normal platelet count (Fig. 1), glycoprotein expression (Table 1) and platelet life span (Fig. 1), showing that CD84 is to a great extent dispensable for platelet production. Lack of CD84 in platelets also did not result in any aggregation or degranulation defect upon stimulation with different agonists (Fig. 2) or a defect in thrombus formation under shear flow conditions (Fig. 4). Further, hemostatic and thrombotic function *in vivo* was unaffected in *Cd84^−/−^* mice (Fig. 5), indicating that loss of CD84 does not affect thrombus stabilization. Similarly, platelet spreading and clot retraction were unaltered (Fig. 3), demonstrating that CD84 is not essential for actin rearrangements in murine platelets. It is conceivable that there is a potential redundancy between platelet adhesion receptors and that the lack of CD84 may be fully compensated. Indeed, a wide range of other receptors have been reported or implicated to modulate platelet-platelet interactions such as ehrins/Eph-kinases [9], JAMs [8], CD150 [10], or SEM4-D [34]. Additionally, soluble mediators are involved in the stabilization of thrombi [6]. On the other hand, our previous finding that CD84 is cleaved from the platelet surface upon platelet activation and aggregation suggests that CD84 may have a different function than stabilizing platelet-platelet contacts. Since besides platelets also many immune cell types abundantly express CD84 and because the receptor undergoes homophilic interactions, it appears possible that the receptor is of functional importance in platelet-immune cell rather than in platelet-platelet interactions. Shedding of CD84 [19] would then provide a potential mechanism to regulate such interactions. However, this potential function of CD84 will be subject of future studies.

Taken together, our results demonstrate that CD84 is not required for proper platelet production and function in hemostasis and thrombosis in mice, strongly suggesting that the receptor is not required to maintain platelet-platelet interactions. Further studies on platelet and immune cell function in *Cd84^−/−^* mice will be an important model to better understand the role of this receptor in thrombotic, inflammatory and/or immunologic processes.
